# Real-time quality control in optoacoustic mesoscopy for enhancing data quality and standardization in clinical studies

**DOI:** 10.1016/j.pacs.2026.100878

**Published:** 2026-08-27

**Authors:** María Begoña Rojas López, Manuel Gehmeyr, Suhanyaa Nitkunanantharajah, Hubert Preißl, Andreas Vosseler, Reiner Jumpertz von Schwartzenberg, Andreas L. Birkenfeld, Nikoletta Katsouli, Nikolina-Alexia Fasoula, Angelos Karlas, Michael Kallmayer, Daniela Branzan, Anette-Gabriele Ziegler, Dominik Jüstel, Vasilis Ntziachristos

**Affiliations:** a Chair of Biological Imaging, Central Institute for Translational Cancer Research (TranslaTUM), School of Medicine and Health & School of Computation, Information and Technology, Technical University of Munich, Munich, Germany; b Institute of Biological and Medical Imaging, Bioengineering Center, Helmholtz Zentrum München, Neuherberg, Germany; c Institute of Computational Biology, Helmholtz Zentrum München, Neuherberg, Germany; d Institute of AI for Health, Helmholtz Zentrum München, Neuherberg, Germany; e German Center for Diabetes Research (DZD), Otfried-Müller-Str. 10, Tübingen 72076, Germany; f Internal Medicine IV, Department of Diabetology, Endocrinology and Nephrology, University of Tübingen, Otfried-Müller-Str. 10, Tübingen 72076, Germany; g Institute for Diabetes Research and Metabolic Diseases (IDM) of the Helmholtz Center Munich at the University of Tübingen, Otfried-Müller-Str. 10, Tübingen 72076, Germany; h Institute of Pharmaceutical Sciences, Department of Pharmacy and Biochemistry, Eberhard-Karls University Tübingen, Auf der Morgenstelle 8, Tübingen 72076, Germany; i Cluster of Excellence EXC 2124 Controlling Microbes to Fight Infections, University of Tübingen, Tübingen, Germany; j M3 Research Center, Malignome, Metabolome, Microbiome, Otfried-Müller-Str. 37, Tübingen 72076, Germany; k Diabetes & Obesity Theme, School of Cardiovascular and Metabolic Medicine & Sciences, Faculty of Life Sciences & Medicine, Kings College London, London, UK; l DZHK (German Centre for Cardiovascular Research), Partner site Munich Heart Alliance, Munich, Germany; m Chair for Computer Aided Medical Procedures & Augmented Reality, Technical University of Munich, Munich, Germany; n Clinic for Vascular Surgery, Helios Klinikum München West, Munich, Germany; o Clinic and Polyclinic for Vascular and Endovascular Surgery, TUM University Hospital, Hospital Rechts der Isar, Technical University of Munich, Munich, Germany; p Institute of Diabetes Research, Helmholtz Zentrum München, German Research Center for Environmental Health, Munich-Neuherberg, Germany; q Forschergruppe Diabetes, Technical University Munich, Klinikum Rechts Der Isar, Munich, Germany; r Munich Institute of Robotics and Machine Intelligence (MIRMI), Technical University of Munich, Munich, Germany

**Keywords:** RSOM, Assessment, Evaluation, SNR, Motion, Cost, Real-time

## Abstract

Raster scan optoacoustic mesoscopy (RSOM) has matured as a medical imaging modality that enables unique high-resolution visualization of optical contrast at depths of several millimeters. Compared with other optical methods, optoacoustics is less affected by photon scattering, enabling superior imaging of dermatological, cardiometabolic, and other conditions. A critical requirement for clinical adoption is the development of methodology that ensures quality control and standardization across subjects, time points, and acquisition environments. We present a machine-learning-based automated real-time quality control method for RSOM using signal-derived metrics for noise and motion. The model was trained and evaluated on 1725 clinical RSOM scans benchmarked against visually perceived image quality ratings from eight experts. The method enables real-time feedback during acquisition to identify suboptimal scans and support standardized high-quality data acquisition. We discuss the impact of the method on clinical RSOM deployment, and the cost benefits achieved through data standardization.

## 1 Introduction

Raster scan optoacoustic mesoscopy (RSOM) is a medical imaging modality that non-invasively visualizes optical contrast in tissues, at depths of a few millimeters, with resolutions in the order of a few tens of micrometers [1]. RSOM enables the non-invasive interrogation of morphological, functional and molecular tissue features at a detail, contrast and depth not available to other optical imaging modalities, that are challenged by photon scattering. Application to human studies has highlighted unique imaging of dermatological conditions [2], including inflammatory burden and treatment efficacy in auto-immune skin disease (psoriasis, eczema) [3], [4], [5], or the ability to visualize melanoma depth and melanoma microvasculature [6]. Applied to systemic conditions, RSOM has characterized diabetes progression or cardiovascular disease using cutaneous disease manifestations [1], [7], [8], [9], [10], [11].

It is now critical to complement this advantageous imaging ability with real-time implementations of quality control (QC) schemes, to mature the RSOM potential for disseminated clinical adoption. Such development can follow the paradigm of other, well-established imaging modalities, such as surgical fluorescence imaging [12], [13], MRI or X-ray imaging [14], [15], [16], [17], which incorporate QC methods to ensure data quality across multiple data acquisition and processing levels [18], [19]. Closed-loop feedback control systems, for example, have been implemented to optimize the outcomes by providing image quality assessment (IQA) in real-time [20], [21], [22].

QC methods have been already considered for RSOM data. A large-scale subjective IQA was conducted by eight qualified observers to benchmark a dataset of 1700 RSOM scans [23]. While subjective IQA is widely regarded as the gold standard [24], [25], [26], it has several shortcomings. It is time-consuming, subjective, as it depends on the individual raters, and its evaluations can be skewed by suboptimal visualization or reconstruction settings [25], [26], [27]. Objective IQA for RSOM was considered in recent studies using indexes that describe motion, measured on the raw RSOM data to offer objective image quality metrics and signal-to-noise ratios (SNR) [28], [29], [30], [31]. In contrast to our preliminary work [31], we here provide a rigorous derivation and definition of the signal-to-noise ratio, along with an extended analysis of the metrics.

In this work we aimed to develop an objective and automated QC method, for real-time quality identification of RSOM scans. The premise of the development is that real-time QC (RTQC) will enable a warning to the operator, in the case of insufficient data quality during scanning, and prompt adjustments, such as better scan-head placement or coupling or system maintenance. To achieve this goal, we adopted a stringent definition of data usability by requiring RSOM images to meet quality levels sufficient for posterior analyses with machine leaning (ML) applications. For simplicity, we refer to the images meeting this criterion as “high quality (HQ)” and those that do not as “suboptimal quality (SQ)”.

We introduce an automated closed-loop RTQC method for RSOM images that uses an objective IQA tool to detect SQ scans and prompt the operator to do an immediate re-scan. The IQA tool utilizes 4 signal metrics computed from electrical noise, laser fluctuations, and vertical motion [28], [31], and outperforms single-metric approaches in predicting image quality of a benchmarked dataset [23]. These classifications were achieved based on raw signal data without requiring image reconstruction, which enabled real-time image quality assessment. Integrating this developed IQA into a closed-loop QC process could increase the probability of acquiring a fit-for-purpose scan for each participant. Importantly, we show in an a priori analysis that the use of the RTQC method can improve on the number of participants required to reach predefined statistical power, by at least 25%, over the current application of RSOM without RTQC.

We discuss the benefits of RTQC not only for alerting operators on sub-optimal scans, but also as a methodology that can be employed in training RSOM sessions, educating less experienced operators on RSOM data quality and on improving their scanning technique.

## 2 Results

### 2.1 RTQC implementation

**Fig. 1:**
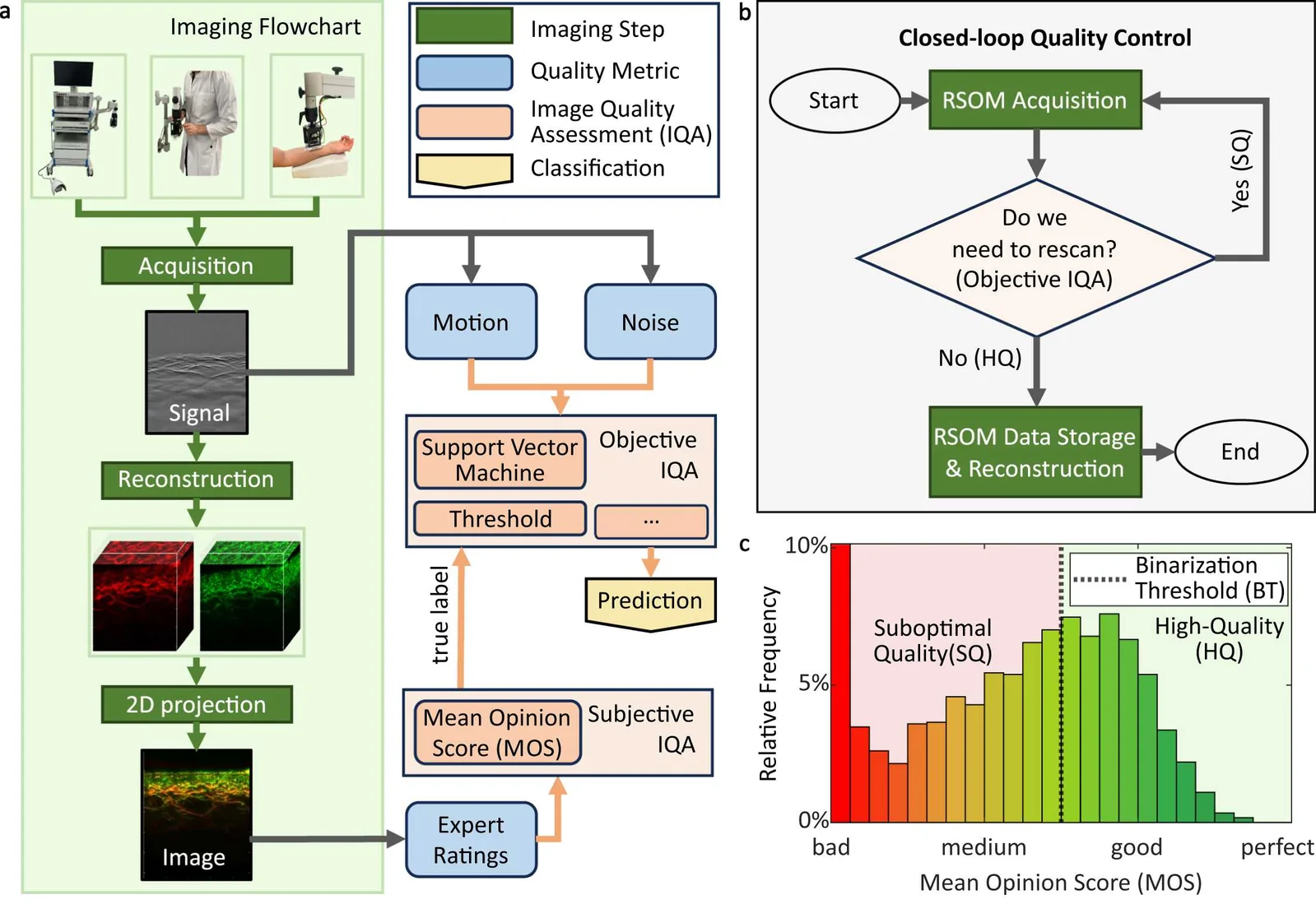
Schematic overview of our quality assessment and quality control procedures for Raster-Scan Optoacoustic Mesoscopy (RSOM) data and the quality threshold used in this study based on subjective expert evaluations. a. Block diagram showing our proposed objective image quality assessment tool (right of green box) integrated into the RSOM imaging workflow (green box) b. Flowchart depicting the closed-loop quality control process using our Image Quality Assessment (IQA) tool. c Histogram of quality labels obtained from subjective expert assessment. These ratings serve as ground truths (true labels in procedure a) for the binary quality classification. The dotted line represents the binarization threshold (BT) used to separate the data into two quality classes: high quality (HQ) and suboptimal quality (SQ).

Fig. 1 maps the methodological steps for developing the objective RTQC in this work, contextualized within the RSOM image process. Each RSOM imaging step, from acquisition to image formation (Fig. 1a, green box), may introduce artifacts that affect the quality of the final reconstructed volumes. Our RTQC method evaluates noise and motion during data acquisition, i.e., two critical factors influencing image quality.

To assess the image quality from raw RSOM data, we sought to define metrics of the raw optoacoustic signal quality that could predict image quality, extending our previous work [31]. Such metrics on the raw signals can enable warning an operator of unsuitable image quality during the scan before reconstructed images are available. We defined four metrics that describe noise and motion:

• the signal-to-noise ratio achieved due to laser fluctuations (SNR_lf_),
• the signal-to-noise ratio achieved due to electrical noise (SNR_en_),
• the mean of the vertical motion estimate (VME_mean_), and
• the standard deviation of the vertical motion estimate (VME_std_).

SNR_lf_ and SNR_en_ quantify two distinct noise sources. SNR_lf_ reflects fluctuations in laser pulse energy, which introduce multiplicative changes in signal amplitude, while SNR_en_ captures additive electronic noise (Methods 4.3.1). Laser energy is monitored with a photodiode [32], and electronic noise is estimated from signal-free regions of the scan. The vertical motion estimate (VME) [28], [29], for details see Methods 4.3.2, captures displacements caused by breathing or probe vibrations. Such misalignments in the raster-scan can introduce distortions in the reconstructed images to the point where anatomical structures become unidentifiable. The metrics VME_mean_ and VME_std_ denote the mean and standard deviation of the VME. The four metrics can be computed in real-time and do not need image formation.

We trained a soft-margin support vector machine (SVM) using these metrics with a third-degree polynomial kernel (“Objective IQA” block in Fig. 1a) to predict RSOM image quality scores assigned expert raters (“Subjective IQA” block in Fig. 1a). The SVM’s predictive performance was validated against other models (see Supplementary Fig. 1 and Supplementary Table 1), where it achieved the best results. The integration of the IQA tool into our closed-loop RTQC framework is illustrated in Fig. 1b. Immediately after completion of each RSOM scan, the acquired data shall be processed, and the quality metrics are computed. If the scan is classified as low quality, immediate feedback can be presented to the operator, prompting a repeat measurement before the participant leaves the imaging session. On a MacBook Pro (M4 Pro, 14-core CPU), quality prediction – including computation of the SNR and motion metrics – takes an average of 1.54 s, while the SVM inference itself requires < 0.01 s and is therefore negligible. As the current implementation performs metric extraction on the CPU, the overall runtime could be further reduced by leveraging GPU-based parallelization.

### 2.2 Objective image quality assessment

RSOM image quality was assessed by eight expert readers. The readers assessed individually the visual quality of 1725 RSOM reconstructed images obtained with two RSOM systems in two clinics [23]. Each scan was rated as low quality (MOS=1), medium quality (MOS=2), high quality (MOS=3), or very high quality (MOS=4). The average quality score of each scan (termed mean opinion score, MOS) became the ground truth image quality scores and the distribution of the MOS values is depicted in Fig. 1c.

To integrate the quality predictor in our closed-loop RTQC such that it could recommend the operator whether rescanning was necessary, we binarized the MOS values by applying a binarization threshold (BT). For this work, we chose a conservative BT – indicated by the dotted vertical line in Fig. 1c – that partitioned the MOS distribution into two categories: high quality (HQ) and suboptimal quality (SQ). Under this scheme, all the scans classified as SQ were flagged for rescanning. This conservative BT was selected empirically to meet stringent quality requirements and to ensure that the accepted data is suitable for downstream applications such as ML-based analysis or other subsequent processing. As a result of this binarization, over 50% of the scans were classified as SQ (Fig. 1c), highlighting the urgent need to improve RSOM data quality for downstream analysis.

**Fig. 2:**
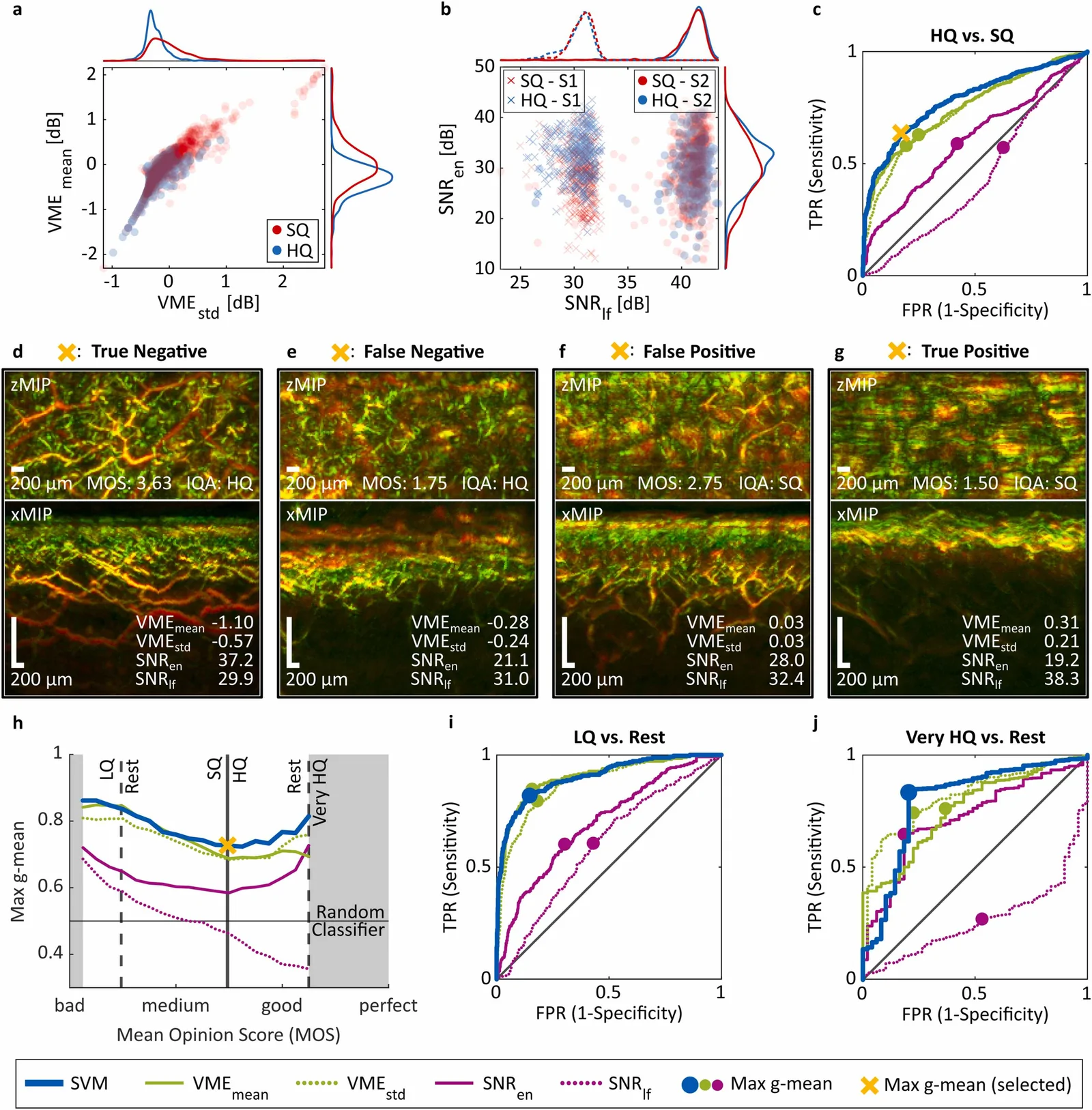
Performance of objective image quality assessment using the proposed Support Vector Machine (SVM) model based on 4 signal metrics, compared to models based on individual metrics. a-b. Distribution of four computed metrics for a random subset (for better visualization) of the data, with each point representing one scan. Images are categorized as suboptimal quality (SQ; red) or high quality (HQ; blue) based on prior subjective classification. Marginal distributions of each quality metric are shown as kernel density plots opposite each axis. b. The plot depicts the differences between two RSOM acquisition systems (System 1, S1 and System 2, S2). S1 and S2 results are indicated by ‘x’ or ‘o’, respectively, on the scatter plot and shown with dashed or solid lines, respectively, in the marginal distribution plots. c. Receiver operating characteristic (ROC) curves showing the predictive performance of the image quality assessment (IQA) models using individual signal quality metrics or the trained SVM model, based on a patient-wise leave-one-out cross validation. The sensitivity and specificity of the optimal classifier decision boundaries, CDB (maximum g-mean, see Methods), are marked with an ‘X’ or a circle for the different classifiers. The ‘X’ identifies the selected classifier subsequently used for reporting results on the unseen test dataset and studying the effects of the quality control procedure. d-g. Representative RSOM images from the test set with different levels of subjective image quality (mean opinion score (MOS) assessed by expert raters), together with the predictions of the selected IQA model (identified by the yellow ‘X’ in (c)) and the corresponding quality metrics. Panels show a true negative, false negative, false positive, and true positive classification, respectively. h. Classification performance of the SVM model and models using single signal metrics across 3 different binarization thresholds of the ground truth labels. LQ: low quality. i,j. ROC curves for classification using LQ or very HQ thresholds as indicated with vertical dotted lines in panel (d). The circles mark optimal decision boundary for each model. VME_mean_: mean of vertical motion estimate, VME_std_: standard deviation of vertical motion estimate, SNR_en_: signal-to-noise ratio based on electrical noise, SNR_lf_: signal-to-noise ratio based on laser fluctuations, FPR: false positive rate, TPR: true positive rate.

The binary subjective quality categories were used to train an objective predictor fed only with the four quality metrics computed on the signal level. These metrics exhibited strong predictive capability for assessing the quality of RSOM images. Fig. 2 depicts the relevance of each metric in predicting image quality and demonstrates the performance of our objective IQA tool.

#### 2.2.1 Motion-related metrics show stronger association with image quality than noise-related metrics on binarized quality categories

The individual metrics for vertical motion estimates (VME_mean_ or VME_std_) demonstrated stronger predictive power for distinguishing between HQ and SQ images compared to the noise-related metrics (SNR_en_ or SNR_lf_). The scatter plots in Fig. 2a and b present the distribution of HQ and SQ scans with respect to the computed signal estimates. Fig. 2a reveals that very low VME_mean_ or VME_std_ values are good indicators for HQ images, whereas high values tend to correspond to SQ scans. However, the VME values alone were not sufficient for reliable classification, as considerable overlap between HQ and SQ samples can be seen in the marginal distributions of Fig. 2a. In contrast, the noise-related metrics (Fig. 2b) exhibited a weaker relationship with visual quality of the RSOM images. SNR_en_ (electrical noise) exhibited a modest correlation with image quality, whereas SNR_lf_ (laser fluctuation) did not show a clear association.

#### 2.2.2 SNR_lf_ reflects system-specific characteristics

While VME_mean_ VME_std_, and SNR_en_ showed comparable mean values across the two systems, with between-system differences small relative to their within-system variability, SNR_lf_ exhibited a pronounced system-dependent difference of approximately 10 dB (Table 1).

**Table 1:** Mean ± standard deviation of the four quality metrics (all reported in dB) computed from the clinical RSOM dataset, stratified by imaging system.

	**VME**_**mean**_	**VME**_**std**_	**SNR**_**en**_	**SNR**_**lf**_
**System 1**	-0.094 ± 0.435	-0.050 ± 0.419	29.9 ± 7.7	30.5 ± 1.3
**System 2**	-0.141 ± 0.341	-0.105 ± 0.241	28.5 ± 9.4	40.8 ± 2.1

As shown in Fig. 2b SNR_lf_ separated the data into two clusters, reflecting different laser fluctuation characteristics of the two RSOM systems used. We additionally measured an identical suture phantom on both RSOM systems. On System 1, repeated measurements acquired over a six-month period yielded SNR_lf_ = 28.72 ± 1.72 dB and SNR_en_ = 41.42 ± 2.15 dB (n = 19). On System 2, measurements acquired on a single day yielded SNR_lf_ = 37.46 ± 0.48 dB and SNR_en_ = 32.33 ± 0.23 dB (n = 3). These measurements reproduced the pronounced difference in SNR_lf_ observed in the clinical dataset, indicating that, in the present dataset, system-specific characteristics dominate the variation of SNR_lf_ compared with image quality. The phantom scans also revealed a difference in the SNR_en_ values, which were not seen in the clinical dataset. While the present study does not allow firm conclusions regarding the origin of this difference, it suggests that SNR_en_ may also contain a system-dependent component, an aspect that warrants further investigation.

#### 2.2.3 Our SVM model outperforms single-metric models in predicting RSOM image quality

The combination of the four signal metrics in an RSOM quality-prediction model achieved superior performance compared with models relying on single metrics. To assess this improvement, the quality estimates produced by our automated objective IQA model – an SVM trained on the four computed metrics – were compared against the predictive ability of each single metric. Fig. 2c presents the receiver operating characteristic (ROC) curves of the SVM model (in blue) and single-metric models (in green and purple), evaluated using a patient-based leave-one-out cross validation approach on the benchmarked dataset. The performance of the single-metric classifiers supports our findings that motion-related metrics have a higher predictive power for image quality than noise-related metrics.

On an unseen test set (N = 345) the SVM achieved a sensitivity of 0.56 and specificity of 0.85 after selecting a classifier decision boundary (CDB; yellow “x” in Fig. 2c). The CDB was chosen by maximizing the geometric mean (g-mean) of sensitivity and specificity on the validation data, providing a balanced operating point. Scans with MOS < 1.5 (i.e., predominantly labeled as “bad”) were correctly classified as SQ with 84% accuracy; in contrast, borderline cases (2 ≤ MOS <3) with only 61% (). These findings highlight the model's strengths in distinguishing "high" and "low" quality scans, with reduced precision for intermediate cases. Omitting the system-dependent metric SNR_lf_ (Section 2.2.2) during SVM training and testing increased the sensitivity slightly to 0.58 but reduced the specificity to 0.76, indicating that SNR_lf_ provides complementary information for classification.

#### 2.2.4 Representative RSOM images highlight the strengths of our SVM model and its limitations for borderline cases

Fig. 2d-g exemplarily show RSOM images projected along the depth (zMIP) and the slow-scanning axis (xMIP) [23], together with their MOS, IQA prediction and corresponding quality metrics. Fig. 2d shows a subjectively HQ scan (MOS 3.625) that was correctly predicted as HQ by the IQA model. The scan exhibits low vertical motion scores and high electrical SNR, consistent with the prediction and underling ground truth. Fig. 2e shows a false negative case. The expert raters assessed the image as below medium quality (MOS 1.75), whereas the IQA model classified the scan as HQ. Despite the comparatively low SNR_en_ (21.1), the low motion-related metric values favored an HQ classification. This suggests that, for this sample, the motion-related metrics had a stronger influence on the model decision than SNR_en_, consistent with their higher predictive power. In contrast, Fig. 2 f shows a false positive case, i.e., an HQ image classified as SQ. With an MOS of 2.75, the scan was rated only slightly above the HQ threshold of 2.5. Similarly, the motion related metrics (VME_mean_ = 0.03, VME_std_ = 0.03) and the SNR_en_ (28.0) fall within an intermediate range between HQ and SQ, with a. tendency to SQ (see marginal distributions in Fig. 2a,b). Thus, this scan represents a typical borderline case in which both the expert rating and the objective quality metrics lie close to their respective decision boundaries, making disagreement between the expert-derived label and the model prediction unsurprising. Finally, Fig. 2 g shows an SQ scan (MOS 1.5) that was correctly classified as SQ, with perceived image quality and objective quality metrics consistently indicating reduced scan quality. In Supplementary Figure 2, we present a broader range of RSOM images, including scans exhibiting motion distortion, poor acoustic coupling, reduced vascular visibility, and excessive noise.

#### 2.2.5 Retrained SVM models achieve the highest performance for extreme image quality classes

In an additional analysis, we investigated how the SVM’s predictive performance varies with the stringency of the image quality definition, modulated by shifting the BT applied to the MOS values. Classification performance peaked when separating very low quality scans from all other scans, reaching a max g-mean of up to 0.86. Similarly, classifying very high-quality scans against the rest also yielded strong performance.

For a systematic assessment, the BT was shifted in 0.125 steps along the MOS range from 1.125 to 3.25. For each BT, the models were retrained (see Methods 4.3.4), and performance (summarized in Fig. 2h) was quantified by the maximum g-mean. Across all thresholds, the SVM achieved comparable or superior performance relative to the individual metrics alone (61% superior by >0.01 points; 39% within ±0.01 points of the best-performing metric). Notably, the lowest accuracy was observed when distinguishing SQ from HQ images, reflecting the subtle and less discernible differences between scans of intermediate quality.

Furthermore, we highlight two BTs, marked with dashed lines in Fig. 2h, which were used to separate LQ (MOS < 1.5) and very HQ (MOS ≥ 2.25) scans from the rest of the dataset. The SVM model demonstrated strong predictive performance for both thresholds, achieving g-mean values of 0.85 for LQ scan detection and 0.81 for very HQ scan detection. The ROC curves for these thresholds are presented in Fig. 2i,j. While vertical motion metrics generally outperformed noise metrics, the electrical noise metric showed comparable importance in identifying very HQ images (Fig. 2j). On the other hand, the laser fluctuation metric functioned as a discriminant feature only when identifying LQ images (Fig. 2i).

### 2.3 A priori cost-saving analysis

**Fig. 3:**
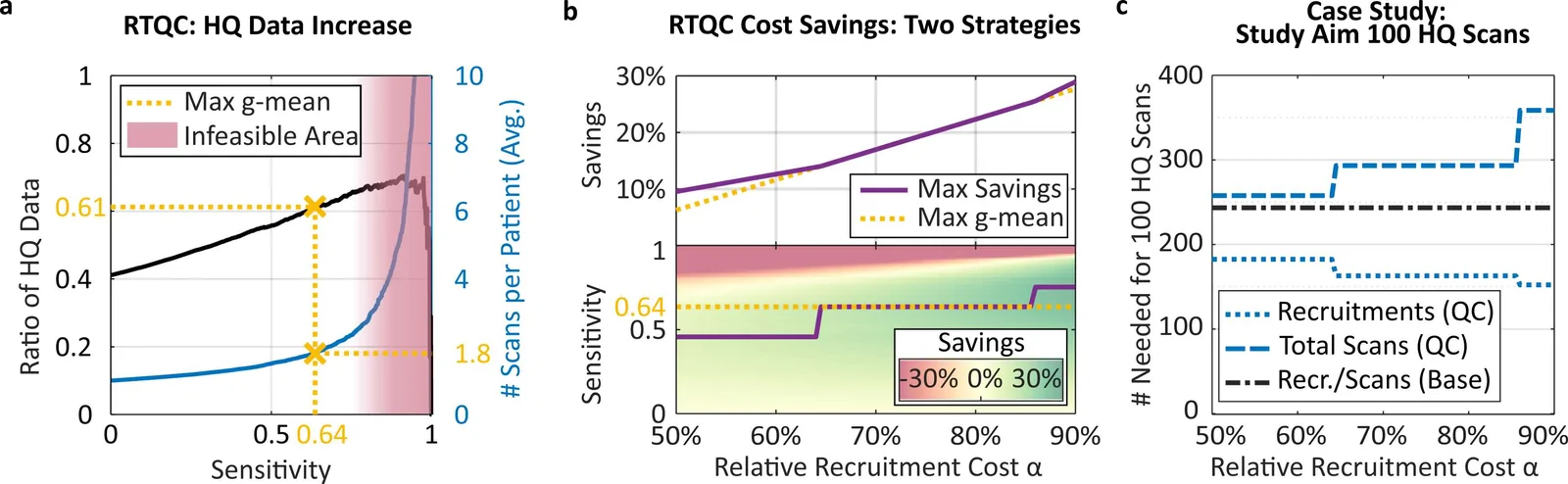
Projected cost savings in Raster-Scan Optoacoustic Mesoscopy (RSOM) clinical studies enabled by the developed data real-time quality control (RTQC) method – an a priori analysis. a. Predicted proportion of high-quality (HQ) data when applying our closed-loop RTQC method. The predicted outcome depends on the selected decision boundary, which sets the sensitivity of the objective image quality assessment (IQA) tool for binary quality classification. Higher detection sensitivity increases the proportion of HQ data but also requires more repeated scans per patient to achieve the target HQ yield. The results corresponding to the maximum geometric mean (Max g-mean) criterion (Fig. 2c) are marked in yellow. b. Estimated recruitment cost savings in clinical studies enabled by our RTQC method. Savings depend on the prediction performance of the underlying IQA model, the selected decision boundary (i.e., sensitivity & specificity on the ROC curve in Fig. 2c), and the relative recruitment cost α (defined as the recruitment cost relative to the total cost of data acquisition). Bottom: Savings plotted as a function of α and IQA sensitivity. Two scenarios for optimizing the IQA are highlighted: Max g-mean in yellow (corresponding to yellow cross in Fig. 2c.) and maximal savings (Max Savings) for a given α in purple. Top: Savings from applying the RTQC tool with different α in the two scenarios. c. Projected number of participants and scans needed when using the RTQC method with the max savings strategy in a case study requiring 100 HQ scans. For comparison, a dotted black horizontal line indicates projections without applying the RTQC tool. ROC: receiver operating characteristic.

We designed a RTQC method that provides real-time feedback during RSOM data acquisition. When our trained IQA tool identifies a scan as SQ, the operator is immediately prompted to repeat the scan. As depicted in Fig. 3, our proposed RTQC process may increase data quality and may reduce the recruitment costs (i.e., less participants needed) at the expense of a higher number of average repeated scans. This trade-off between the repeated scans and the required number of participants can be adjusted by tuning the CDB (see Methods 4.4.1) to achieve an optimal sensitivity-specificity balance along the ROC curve of the IQA (see Fig. 2c).

#### 2.3.1 Our data quality control method may increase the proportion of HQ images and will may decrease clinical study costs

Fig. 3a depicts how the proportion of HQ images and the average number of repeated scans per patient vary with sensitivity, which is controlled by the CDB on the ROC curve. Using the maximum g-mean criterion (see Methods 4.3.3) for CDB selection (yellow “x” in Fig. 3a and Fig. 2c), the proportion of usable HQ images would increase from 0.41 to 0.61. This improvement corresponds to an average of 1.8 scans per patient.

To quantify this trade-off, we performed a projected cost analysis (see Methods 4.4.2). Applying our RTQC tool during a clinical study can lead to potential cost reductions for data acquisition of 5–30% compared to a study conducted without RTQC (referred to as baseline in this work). The extent of these savings depends on four key factors:

• The baseline HQ yield of the imaging system, i.e., the negative rate (fixed at $p_{N}=0.41$ for current RSOM systems),
• The performance of the SVM-based IQA classifier (derived from the ROC curve),
• The adjustment of the CDB along the ROC curve, and
• The relative recruitment cost α, defined as the ratio of recruitment cost within the total per-scan cost (scanning + recruitment).

We explored two scenarios for choosing the CDB:

1. Max g-mean: selecting the CDB that maximizes the g-mean (see Methods 4.3.3), or
2. Max savings: selecting the CDB that maximizes cost savings (see Methods 4.4.3 & 4.4.4).

The exact cost savings depend on α which varies between studies. We analyzed both scenarios across a broad range of plausible α values. For reference, α=50% indicates equal scanning and recruitment costs, while an α=90% means that recruitment is nine times more expensive than scanning. As visualized in Fig. 3b, both scenarios resulted in identical CDBs, when α ranged between 65% and 85%, corresponding to recruitment cost approximately 2–6 times higher than scanning costs. We consider this a realistic range for α, as recruitment is usually much more expensive than scanning. This finding indicates that the max g-mean criterion can serve as a generally applicable and robust strategy for selecting the CDB, offering a good balance between performance and cost savings.

#### 2.3.2 Case study for a clinical study design shows potential cohort reductions due to RTQC

In a case study requiring 100 HQ scans, the increase in HQ yield enabled by the RTQC tool would reduce the necessary cohort size from 244 to 163 participants (33% reduction), while increasing the total number of scans from 244 to 294 (20% increase).

Fig. 3c extends this analysis by examining how both the total number of scans and the required number of participants change as a function of the relative recruitment cost α, assuming the RTQC tool is applied and the CDB of the IQA is optimized for cost savings (Scenario 2). Depending on how cost is distributed between requirement and acquisition, the required cohort ranges from 183 (25% reduction at α=50%) to 152 participants (38% reduction at α=90%). In parallel, the total number of scans ranges from 258 (6% increase at α=50%) to 359 (47% increase at α=90%).

## 3 Discussion

This work presents the RTQC as a method for performing QC on RSOM data in real-time during data acquisition. The method operates in a closed-loop process, prompting operators for insufficient scan quality. Such warning can result in readjusting the placement of the device, check the operational parameters or prompt maintenance in the case of malfunction. Data quality is objectively assessed using an IQA tool based on a trained SVM model, which is incorporated into the RTQC tool. We found that the SVM model accurately predicts image quality labels without requiring image reconstruction. Additionally, we demonstrated in an a priori analysis that implementing our RTQC method in clinical studies could lead to substantial cost savings by decreasing the required cohort size while maintaining the amount of usable data for a defined study objective. While individual objective quality control metrics and scores for RSOM have previously been proposed, to the best of our knowledge, this is the first solution for RTQC validated on a large RSOM image dataset. The ability to evaluate image quality during acquisition has the potential to enhance the usability of RSOM and reduce costs in clinical studies, thereby accelerating its adoption and translation into clinical practice.

The IQA tool utilizes ML techniques to assess image quality based on four metrics that capture physical factors known to degrade RSOM scans. Although subjective assessment remains the gold standard in IQA [24], [26], it has major drawbacks including high time demands, considerable costs, and variability in scores across different raters or for a single rater over time (inter and intra-expert variability). Our IQA tool (SVM model) addresses these challenges by providing an automated and objective solution predicated on quality indices based on motion and SNR [28], [30], [31], [33]. The developed objective IQA tool has been rigorously validated using a large dataset of RSOM images (N = 1725) annotated with quality labels assigned by 8 experts [23]. Evaluation against this expert-labeled “ground-truth” dataset showed that our objective IQA tool achieved high accuracy in identifying data of either low or very high quality (extreme BTs). While the tool had some difficulty differentiating subtle variations at intermediate quality levels, it can accurately identify HQ images, which is essential for ensuring suitability of data collected for almost all downstream applications.

The IQA tool assesses the quality of RSOM images by evaluating noise and motion in the recorded data, which are the primary sources of artifacts in OptA imaging modalities [27], [28], [29], [34], [35]. A detailed analysis revealed that motion has a greater impact on image quality than noise (see Results 2.2.1). Nonetheless, including all four metrics related to noise and motion in the IQA tool increased overall evaluation performance. This tool advances beyond prior work [30], which required pre-selection of images with high SNR to prove the prediction ability of the noise-quality-index. SNR is typically computed on reconstructed images rather than raw signal data and has proven to be a useful quality indicator in modalities, such as OptA and magnetic resonance tomography [36], [37], particularly for purposes like assessing denoising methods [38]. In contrast, our SVM model assesses quality using inputs calculated in the signal domain without needing image reconstruction. Consequently, our IQA tool can rate quality in real time, distinguishing it from previous objective and subjective quality assessment methods. Nonetheless, since signal-domain metrics cannot fully capture all factors influencing the visual quality of reconstructed images, our IQA tool has some inherent limitations. To tackle these limitations, we implemented measures to minimize assessment bias, such as employing standardized image flattening, contrast adjustments [23], and averaging evaluations across eight independent experts. Despite these existing limitations, our IQA tool demonstrates reliable real-time detection of images with insufficient quality with high accuracy. In the future, quality prediction accuracy can further be improved by leveraging advanced ML techniques. For example, fully deep-learning no-reference IQA methods [39], [40] show considerable promise in modeling more complex relationships between signal characteristics and image quality.

An important finding of this study is the pronounced system dependence of the laser-fluctuation metric SNR_lf_. Despite both RSOM systems being shipped at the same time and operated with identical hardware (including the same laser source and transducer), software configuration, and acquisition protocol, SNR_lf_ consistently separated the data according to imaging system. Neither the documented system configuration nor the routine calibration measurements provided a sufficient explanation for this behavior (Section 4.1.3), and the underlying cause of these system-specific characteristics remains to be elucidated. Although SNR_lf_ exhibited the strongest system dependence of the four quality metrics, excluding it from the feature set resulted in a slight reduction in classification performance, indicating that it contributes complementary information for IQA rather than merely encoding system identity.

A potential approach for cross-system calibration is the use of standardized phantoms, such as a simple suture phantom. In particular, system-specific biases in SNR_en_ could be characterized and accounted for using standardized phantoms. In contrast, phantoms are of limited value for calibrating the motion-related metrics, as these primarily reflect external and physiological motion during clinical imaging, such as patient movement and blood circulation, which cannot be realistically reproduced using physical phantoms. Nevertheless, standardized tissue-mimicking phantoms could serve as valuable tools for the qualitative and quantitative calibration and harmonization of RSOM systems across clinical sites, extending beyond RTQC and quality metric calibration. However, developing tissue-mimicking phantoms with sufficient long-term stability for longitudinal quality assurance remains challenging, particularly in optoacoustic imaging, where both optical and acoustic properties must be reproduced [41], [42]. Future work should therefore investigate whether standardized tissue-mimicking phantoms can facilitate cross-system calibration and improve the transferability of RTQC models.

Beyond hardware-specific considerations, like all ML-based approaches, our IQA tool may be sensitive to out-of-distribution data [43], [44], meaning that it could yield unexpected results under unusual scanning situations or when acquisition settings change substantially, such as variations in coupling conditions, operator experience, room temperature or hardware-specific characteristics. To mitigate this limitation, we evaluated the model on an unseen subset of the data, showing that the prediction performance showed only minimal decline. Nevertheless, this highlights the need for further improvements to enhance the tool’s adaptability and generalizability to new scenarios. Additionally, the developed IQA tool is task-specific and designed for binary classification to determine whether acquired data meets a predefined threshold for fit-for-purpose. Hence, establishing an appropriate quality threshold (i.e., a BT) is essential and can be non-trivial. This means that some expertise is needed to select the appropriate IQA tool or re-train it when assessing image quality for new applications. In particular, device-specific variations, as observed for the laser-fluctuation metric, may affect model transferability. Likewise, incorporating a certain number of quality-rated scans acquired on a new device or under a different acquisition protocol may facilitate adaptation to previously unseen system-specific characteristics.

Given that recruitment costs constitute a major fraction of the total clinical trial costs [45], [46], it is important to evaluate the potential cost savings enabled by our RTQC tool. A preliminary analysis indicated that the closed-loop feedback RTQC can reduce recruitment costs by up to 30% by decreasing the number of participants needed to obtain a predefined amount of high-quality data and to meet the study goal. In addition, the RTQC tool supports operators in consistently acquiring high quality data by providing immediate prompts when a re-scan is needed. This real-time feedback reduces the reliance on the operator’s expertise in RSOM and can be further used in operator training [47]. These cost projections are nevertheless subject to certain caveats. Calculating the optimal classifier threshold for the RTQC tool to reap maximum cost savings requires assumptions about parameters concerning relative recruitment cost α, the current underlying average data quality, and the prediction performance of the IQA (ROC curve). If these assumptions change, the projected savings and the strategy for selecting the cost optimal CDB of the IQA classifier must be recalculated in accordance with the methodology presented in this study. In contrast, selecting the CDB by maximizing the geometric mean (g-mean) depends solely on the ROC curve and is therefore broadly applicable without being affected by the other assumptions mentioned above.

In general, finding a balance between sensitivity and specificity is a complex challenge across scientific disciplines. In a comparable setting [48], where signal quality assessment methods for physiological monitoring systems were evaluated, the authors claimed that high sensitivity to exclude unreliable data should be prioritized over high specificity, given that signal acquisition is nearly cost free (α≈1) in a monitoring framework. However, in OptA mesoscopy, the trade-off between repeating scans and inadvertently accepting low-quality signals is more complex due to the higher costs associated with repeated scans (α<1). This complexity necessitates a more nuanced optimization approach, as implemented through the a priori RTQC cost analysis presented in this study. It is important to note that the cost analysis of the RTQC tool is not tied to OptA mesoscopy and can be applied to any system employing closed-loop feedback QC frameworks, such as other medical devices, industrial production lines, or any system requiring iterative quality assessment and feedback mechanisms.

Overall, the developed RTQC and IQA methods for RSOM data realized necessary steps toward the broader adoption of OptA mesoscopic imaging in clinical settings. The methodology for setting thresholds for quality assessment using the RTQC tool and projecting costs can be deployed to guide design of future RSOM clinical studies. Our methodology enables determination of the minimum cohort size needed to achieve a target number of high-quality fit-for-purpose scans based on established SNR and motion scores. Another consideration is that the clinical status (e.g., diseased versus healthy skin) or skin type might affect the images’ visual quality, as previously discussed in [23]. Thus, further investigation is needed to evaluate the performance of the prediction model for diseased skin and assess if there are biases related to different skin types. Despite this limitation, we believe that the work presented here represents a crucial step toward implementing quality assurance methods for OptA mesoscopy, which is especially important given the increasing emphasis on in-depth medical data analysis [18], [19], [49]. Our proposed RTQC method could be integrated to trigger reactive measures as part of a larger framework for quality assurance and management for RSOM systems [50], [51]. Indeed, integration of the proposed RTQC framework is planned for the next generation of RSOM systems.

RSOM is a generally recent imaging modality [52], [53], that is now gaining attention for clinical application [2], [9], [54]. In this work, we show a next stage in the development of this modality, by automating the real-time quantification of RSOM data quality and present the benefits of implementing quality control during acquisition. The RTQC method can improve the technology maturity and potential for clinical adoption by making RSOM data acquisition user-friendly and, possibly, reducing the cohort size needed to achieve a predefined number of high-quality scans in clinical studies. This advancement paves the way towards broader clinical adoption of OptA mesoscopy, allowing the healthcare community to benefit from its unique capabilities and features, which enable applications not attainable with current clinical imaging modalities.

## 4 Methods

### 4.1 RSOM data

#### 4.1.1 RSOM clinical studies: origin of data

The data analyzed in this manuscript were acquired within the framework of three clinical studies investigating the impact of diabetes and cardiovascular disease on skin microvasculature. The studies were conducted at the TUM University Hospital (Hospital Rechts der Isar), Technical University of Munich (Germany), and at the University Hospital Tübingen (Germany).

Measurements were performed using two RSOM Explorer C50® research systems (from iThera Medical GmbH, now under Spear UG, Munich Germany). In total, 1725 RSOM scans were collected from 236 participants (Munich: N = 174; Tübingen: N = 62). Imaging was conducted at the inner forearm during cuff occlusion and release (7–13 scans per participant) and at the pretibial region of the lower leg (1–3 scans per participant). Further details regarding study design, inclusion criteria, imaging protocol, and preprocessing procedures are described in our previous publication [23].

#### 4.1.2 RSOM imaging systems

Both systems were shipped to the respective clinics in April 2021 with identical hardware and software configurations, including the same transducer (HFM31, Sonaxis), laser source (FDSS532-Q3, CryLas), and software version. The data used in this study were acquired between May 2021 and February 2023 at Tübingen and between May 2021 and November 2022 at TUM. During the study period, maximum pulse energy was recorded as part of routine six-month calibration and service procedures. Across all service measurements, the average pulse energy was 11.22 μJ for System 1 and 12.83 μJ for System 2, with mean relative standard deviations of 0.27% and 0.23%, respectively.

#### 4.1.3 RSOM scanning procedure

The RSOM C50 systems recorded the optoacoustic response of illuminated tissue within a 4 × 2 mm scanned area at a sampling rate of 500 MHz. The time-recorded signals, also called A-lines, were distributed along a raster path over the scanned area in steps of 0.015 mm.

#### 4.1.4 RSOM raw signals

RSOM data in the form of raw time-resolved signals were used to compute the metrics for quality assessment. The raw data were pre-processed by bandpass filtering the recorded signal over the scanning area (also known as sinogram) between 10 and 99 MHz. The broad bandwidth was selected based on the frequency specifications of the utilized transducers.

### 4.2 Visual IQA by experts

#### 4.2.1 Benchmark dataset

A subjective IQA was performed on 1725 RSOM images to obtain the ground truth quality labels for the dataset. A panel of eight experts evaluated the visual quality of the volumetric reconstructions to establish a benchmark dataset [23]. All raters were researchers in the field of optoacoustic imaging with between one and seven years of experience in optoacoustic image acquisition, reconstruction, and/or analysis at the time of the study. The dataset comprised of annotated RSOM images created using the enhanced maximum intensity projection (eMIP) method. eMIP mitigates biases during subjective quality assessment by automatically detecting and correcting curved skin surfaces, thereby enhancing the image clarity by preventing overlaying of features from varying skin depths. Additionally, eMIP improves dynamic contrast adjustment, enabling clear differentiation between smaller and larger features, corresponding to high and low frequencies, respectively, by assigning two different colors to represent different superficial layers of the skin. The maximum intensity projection (MIP) images of the benchmark dataset were rated by experts on a scale of 1 (=bad quality) to 4 (=perfect quality), taking into account the usability of the data for future data analysis. The MOS for each image was calculated by averaging the score given by the 8 evaluators.

#### 4.2.2 Binarization of quality scores

For maximal ease of use, especially for less experienced operators, a RTQC tool should provide straightforward binary classification indicating whether the data quality is sufficient or insufficient. A total of 20 binarization thresholds, ranging from 1 to 4 in steps of 0.125, were considered. Based on this assessment, three specific thresholds were selected to identify data with sufficient quality for different downstream applications. For example, a threshold of 2.5 was set to identify high-quality (HQ) data suitable for ML-based data analyses, with data falling below this threshold classified as suboptimal quality (SQ). If SQ data is acquired, the RTQC tool would immediately prompt an operator to repeat the scan. Additionally, thresholds of 1.5 and 3.25 were introduced to distinguish very low-quality data or very high-quality, respectively, for mimicking different quality standards for subsequent data analysis.

### 4.3 Objective IQA process

The binarized labels (SQ or HQ) assigned by experts served as the ground truth for the data quality assessment method. The method included the two elements below:

• **Predictors**: These inputs for the classifier included SNRs due to electrical noise (SNR_en_) or laser fluctuations (SNR_lf_), as well as vertical motion estimates (VMEs), which relate to noise and motion in the raw data.
• **Prediction Model:** This supervised classifier predicts the data’s quality (e.g., SQ or HQ) using the calculated predictor values and the binarized MOS values as ground truth labels.

#### 4.3.1 Signal-to-noise ratio

##### 4.3.1.1 SNR definitions and noise model

There is no single uniform definition of SNR or contrast-to-noise ratio (CNR) for signals acquired by medical devices, and multiple definitions have been reported in literature [55], [56]. In this study, the SNR (in decibels [dB]) of a noisy, filtered sinogram $S_{n}$ is defined as the square of the peak ground truth signal S over the power of the noise N

(1) $$\mathrm{SNR}\left[S_{n}\right]=10\log_{10}\frac{\mathrm{peak}{\left[S\right]}^{2}}{P[N]},$$

where the power operator is defined by $P\left[\cdot \right]:=E\left[\;\cdot^{2}\right]=\int x^{2}\;\text{d}\cdot (x)$. The noisy signal $S_{n}$ is modeled by

(2) $$S_{n}=S{\cdot N}_{\mathrm{lf}}+\;N_{\mathrm{en}},$$

where the electrical noise $N_{\mathrm{en}}$ and the laser fluctuations $N_{\mathrm{lf}}$, are additive and multiplicative noise terms, respectively. We assume that electrical noise, laser fluctuation and ground-truth signal are independent. Furthermore, we assume that the electrical noise (after pre-processing) follows a normal distribution with zero-mean and standard deviation $\sigma_{\mathrm{en}}$, whereas the laser fluctuations, after normalization, follows a normal distribution with a mean of 1 and a standard deviation of $\sigma_{\mathrm{lf}}$. The noise is defined as the difference between the noise signal and the ground truth signal,

(3) $$N≔S_{n}-S=S\left(N_{\mathrm{lf}}-1\right)+N_{\mathrm{en}}.$$

The power of the total noise in the signal can be expressed as

(4) $$P\left[N\right]=P\left[N_{\mathrm{lf}}\right]+P\left[N_{\mathrm{en}}\right]=E\left[S^{2}\right]∙\sigma_{\mathrm{lf}}^{2}+\sigma_{\mathrm{en}}^{2},$$

where $P\left[N_{\mathrm{lf}}\right]=E\left[S^{2}\right]\cdot \sigma_{\mathrm{lf}}^{2}$ represents the power of the noise coming from the laser fluctuation, and $P\left[N_{\mathrm{en}}\right]=\sigma_{\mathrm{en}}^{2}$ represents the portion coming from the electrical noise.

##### 4.3.1.2 Estimation of random variables

The RSOM system records a portion of the applied illumination using a photodiode [32], providing a reference for the energy used during optical excitation and its variability across pulses. Assuming the photodiode measurements follow a normal distribution $L∼N(\mu_{L},{\;\sigma }_{L})$, we can approximate the standard deviation of the laser fluctuations by $\sigma_{\mathrm{lf}}^{2}=\;\frac{\sigma_{L}^{2}}{\mu_{L}^{2}}$. The electrical noise ${P\left[N_{\mathrm{en}}\right]=\sigma }_{\mathrm{en}}^{2}$ is estimated using the first 50 time-samples of all recorded A-lines, where no signal is expected. The power of the noisy signal $P\left[S_{n}\right]=E[S_{n}^{2}]$ is calculated from all data points.

##### 4.3.1.3 SNR estimation for laser fluctuation and electrical noise

Breaking down SNR according to the two noise terms $N_{\mathrm{fl}}$ and $N_{\mathrm{en}}$ allows for the derivation of two separate SNR formulas $\mathrm{SN}R_{\mathrm{lf}}$ and $\mathrm{SN}R_{\mathrm{en}}$. These two SNR values serve as quality metrics or features in the objective IQA (SVM). The SNR with respect to the laser fluctuation is given by

(5) $${\mathrm{SNR}}_{\mathrm{lf}}\;\left[S_{n}\right]=10\log_{10}\frac{\mathrm{peak}{\left[S\right]}^{2}}{P\left[N_{\mathrm{lf}}\right]}=10\log_{10}\frac{1}{\sigma_{\mathrm{lf}}^{2}},$$

and the SNR with respect to the electrical noise by

(6) $$\mathrm{SN}R_{\mathrm{en}}\left[S_{n}\right]=\;10\log_{10}\frac{{\mathrm{peak}\left[S\right]}^{2}}{P\left[N_{\mathrm{en}}\right]}=10\;\log_{10}\frac{P\left[S\right]}{\sigma_{\mathrm{en}}^{2}}+10\log_{10}\delta^{2},$$

whereδ is a parameter that needs to be set to approximate the peak power properly (see Methods 4.3.1.4). In (5), (6), the second identity follows from the robust peak signal estimation, $\mathrm{peak}\left[S\right]$, in Methods 4.3.1.4. For completeness, we have provided the equation used for calculating the combined SNR below, although it was not used as an independent quality metric in this paper

(7) $$\mathrm{SNR}\;\left[S_{n}\right]=-10\log_{10}\left(\sigma_{\mathrm{lf}}^{2}+\frac{\sigma_{\mathrm{en}}^{2}}{\delta^{2}E\left[S^{2}\right]}\right).$$

##### 4.3.1.4 Estimating peak signal robustly using signal power

The peak amplitude estimated by the maximum over all received data points is highly sensitive to outliers, as an RSOM scan can consist of over 50000 single A-lines, making it a non-robust estimation of the peak signal, $\mathrm{peak}\left[S\right]$, for our RSOM scans. Empirically, we found that the peak amplitude of a signal is approximately δ=7 standard deviation above the noisy signal S for the data considered in this manuscript. Therefore, the following formula is proposed to robustly estimate the peak amplitude,

(8) $$\mathrm{peak}{\left[S\right]}^{2}=N^{2}E\left[S^{2}\right]=N^{2}\;\frac{E\left[S_{n}^{2}\right]-\sigma_{\mathrm{en}}^{2}}{1+\sigma_{\mathrm{lf}}^{2}}=\delta^{2}P\left[S\right],$$

where we used the following two identities

(9) $$S=\frac{S_{n}-N_{\mathrm{en}}}{N_{\mathrm{lf}}},$$

(10) $$P\left[S\right]=E\left[S^{2}\right]=\;\frac{E\left[S_{n}^{2}\right]{-\sigma }_{\mathrm{en}}^{2}}{1+\sigma_{\mathrm{lf}}^{2}}.$$

#### 4.3.2 Vertical motion estimate

In the RSOM raster-scan, individual scans (A-lines) are acquired rapidly along the fast-scanning axis, incrementally advanced along the slow-scanning axis; each sweep along the fast-scanning axis is termed B-plane. Due to this temporal sequencing, motion along the fast-scanning axis is typically much smaller than along the slow-scanning axis. Hence for computational efficiency, vertical motion scores are only calculated between subsequent B-planes as presented in [28]. The metrics VME_mean_ and VME_std_ represent the base 10 logarithm of mean and standard deviation of all VME indices, respectively.

#### 4.3.3 Prediction model

For objective and automated IQA, 4 signal metrics of interest (SNR_en_, SNR_lf_, VME_mean_ and VME_std_) were computed from the filtered raw sinogram of each scan, as previously described. Given these inputs, the IQA model was trained to predict the binarized MOS (see Methods 4.2.2) for each scan. Based on the chosen CDB (i.e., the decision boundary), the model outputs a binary quality label, such as HQ or SQ.

The dataset of 1725 scans was split into a training (80%) and test (20%) set using balanced stratified sampling. We ensured that the split preserved the proportion of scans coming from the two RSOM systems and that the average MOS remained comparable between the sets. To prevent data leakage, all scans from a given patient were assigned to either the training or test set.

The training set was used to select the best performing model. Candidate models were validated using patient-wise leave-one-out cross-validation, where all scans from a single patient were excluded in each fold for validation. For each validated scan, the posterior probability p was collected. The binary labels SQ or HQ are assigned when p is larger or smaller than a value, $p_{\mathrm{th}}$ (i.e., $p<p_{\mathrm{th}}$ or $p\geq p_{\mathrm{th}}$*)*. An ROC curve was then constructed by varying the decision boundary $p_{\mathrm{th}}\;$applied to these posterior probabilities. We computed the geometric mean (g-mean) of sensitivity and specificity for all decision boundaries along the ROC curve. The classifier’s optimal CDB was defined as the threshold corresponding to the sensitivity-specificity pair that maximizes the g-mean, ensuring a balanced trade-off between false positives and false negatives. Furthermore, we used the maximum g-mean as the metric to identify the best-performing classifier. The performance of the final model was evaluated on an unseen test set used exclusively for this purpose. Throughout this paper, SQ scans are treated as the positive class.

The best performing classifier was a soft-margin SVM with a third-degree polynomial kernel, trained on standardized input data. An asymmetric misclassification cost was applied to account for imbalanced data. The cost of a false positive was fixed at 1, while the cost of a false negative was set to the ratio of the negative to positive class frequencies. Details of all other candidate classifier models are presented in Supplementary Materials 1.1.1.

For comparison, individual metrics were evaluated against the SVM model using a simple threshold-based classifier. To enable a fair comparison, metric values were first normalized and then mapped to probabilistic posterior outputs using the sigmoid function, facilitating consistent ROC-based performance evaluation. The same patient-wise leave-one-out cross validation procedure was applied to construct the ROC curves, with the CDB derived following the same g-mean criterion.

#### 4.3.4 Quality assessment with different quality thresholds for binary classification of the benchmark dataset

Different SVM models were retrained using the kernel presented before using different binarization of the data beyond HQ and SQ (i.e., changing the BT). Specifically, we applied a total of 18 different BTs (from 0 to 2.25 in steps of 0.125) to the MOS. For each BT, we performed a patient-wise leave-one-out cross-validation across the different classification models. For comparison reasons, we also evaluate the threshold classifier on the individual metrics for the different BTs.

### 4.4 Analysis of the quality control method

We integrated the developed IQA, based on an SVM (Methods 4.3.3), into a closed-loop data QC method. In this RTQC method, the quality predicted by the IQA is “fed back” as input during the scanning process: if suboptimal quality (SQ) data is detected, the RTQC tool immediately prompts the operator to repeat the measurements. Leveraging the trained SVM, our proposed data RTQC method is capable of operating in real time during each acquisition directly on the recorded signal data. In the following section, we present an analytical, a priori derivation of the consequences of applying our RTQC method. The derivation stands alone and can be applied to any underlying classifier with an ROC curve and adjustable CDB.

#### 4.4.1 Expected number of measurements needed to obtain data with adequate quality

When RTQC is applied, measurements labeled as SQ by the IQA are repeated. These SQ labels can be true positives (TP; images correctly classified as SQ) or false positives (FP; HQ images wrongly classified as SQ). Repeated measurements may also be labeled as SQ again, requiring further repetitions until a HQ classification is achieved. This process continues until a measurement is finally labeled as HQ and is not rejected. The expected number of measurements to obtain one HQ scan can therefore be quantified by

(11) $$E\left[\text{\#scans}\right]=\sum_{j=0}^{\infty }{\left(p_{\mathrm{TP}}+p_{\mathrm{FP}}\right)}^{j}=\frac{1}{p_{\mathrm{TN}}+p_{\mathrm{FN}}}=\frac{1}{p_{P}\left(1-\mathrm{sens}\right)+p_{N}\mathrm{spec}},$$

with the probability variables defined in Table 2. If no RTQC is applied, $E\left[\mathrm{\#scans}\right]=1$; which is equivalent to the tuple (sens=0,spec=1). When RTQC is applied, the expected ratio of usable HQ data can be quantified by

(12) $$E\left[\mathrm{usability}\right]=p_{\mathrm{TN}}\sum_{j=0}^{\infty }{\left(p_{\mathrm{TP}}+p_{\mathrm{FP}}\right)}^{j}=\frac{p_{\mathrm{TN}}}{p_{\mathrm{TN}}+p_{\mathrm{FN}}}=\frac{p_{N}}{p_{P}LR^{-}+p_{N}}.$$

**Table 2:** Definition and description of probability variables. SQ: suboptimal quality, HQ: high quality.

$p_{\mathrm{TP}}$	true positive rate (probability of images correctly classified as SQ)	$p_{P}$	positive rate (probability of SQ scan ($p_{\mathrm{TP}}+p_{\mathrm{FN}}$))
$p_{\mathrm{TN}}$	true negative rate (probability of images correctly classified as HQ)	$p_{N}$	negative rate (probability of HQ scan ($p_{\mathrm{TN}}+p_{\mathrm{FP}}$))
$p_{\mathrm{FP}}$	false positive rate (probability of images wrongly classified as SQ)	sens	sensitivity (probability of classifying image as SQ given that the image is SQ)
$p_{\mathrm{FN}}$	false negative rate (probability of images wrongly classified as HQ)	spec	specificity (probability of classifying image as HQ given that the image is HQ)

The function is strictly decreasing with respect to the negative likelihood ratio $LR^{-}≔\frac{1-\mathrm{sens}}{\mathrm{spec}}.$ If no RTQC is applied, $E\left[\mathrm{usability}\right]=p_{N}$. If the scans identified as SQ by the RTQC tool (positive class) are not discarded - allowing potential false positives (i.e., actual HQ scans mistakenly flagged as SQ) to be retained in the data set, the calculation for $E\left[\mathrm{usability}\right]$ must be adapted as described in Supplementary Materials 1.1.2.

#### 4.4.2 Cost benefits of applying the quality control tool

In a study where a target number of HQ images, $N_{\mathrm{target}}$, must be collected to test a hypothesis, the probability of obtaining an HQ image from a single measurement without RTQC is denoted by $p_{N}$. Hence, the expected number of patients that need to be recruited to achieve $N_{\mathrm{target}}$ HQ images is

(13) $$N_{\mathrm{recr}}=\frac{N_{\mathrm{target}}}{p_{N}}.$$

The overall cost to obtain $N_{\mathrm{target}}$ HQ images can be split into recruitment costs and scanning costs. These costs may be interpreted in monetary terms (e.g., in euros or dollars), but it can also be conceptualized more broadly to include non-monetary burdens, such as physical discomfort or psychological strain. The average cost associated with measuring a patient (scanning cost) is denoted by $c_{\mathrm{scan}}$, which includes only costs directly related to the scanning procedure, including both monetary and, where applicable, non-monetary components. This may include costs from wear of the device and any non-monetary extra burden placed on the patient. The average recruitment cost, denoted by $c_{\mathrm{recr}}$, encompasses all other costs, including overhead costs for writing ethics protocol, individual patient-related costs like carrying out informed consent and any costs incurred in preparing for the measurements, such as setting up and calibrating the system. The overall cost is then divided by the number of recruited patients $N_{\mathrm{recr}}$. For simplicity, we assume that $c_{\mathrm{recr}}$ remains constant regardless of $N_{\mathrm{recr}},$ therby ignoring any scaling effects.

The total base cost $c_{\mathrm{base}}\;$(cost without RTQC) to acquire all the data for the study is

(14) $$c_{\mathrm{base}}=N_{\mathrm{recr}}\left(c_{\mathrm{recr}}+c_{\mathrm{scan}}\right)=N_{\mathrm{target}}\frac{c_{\mathrm{recr}}+c_{\mathrm{scan}}}{p_{N}}.$$

When RTQC is applied, the total number of HQ data points can be estimated by $E[\mathrm{usability}]\cdot N_{\mathrm{recr}}$,. Given a target of $N_{\mathrm{target}}$, this can be expressed as

(15) $$N_{\mathrm{recr}}=\frac{N_{\mathrm{target}}}{E[\mathrm{usability}]}=N_{\mathrm{target}}\frac{p_{\mathrm{TN}}+p_{\mathrm{FN}}}{p_{\mathrm{TN}}}.$$

Furthermore, the expected total number of measurements is given by

(16) $$N_{\mathrm{scan}}=E[\#\mathrm{scans}]\cdot N_{\mathrm{recr}}=\frac{N_{\mathrm{recr}}}{p_{\mathrm{TN}}+p_{\mathrm{FN}}}=\frac{N_{\mathrm{target}}}{p_{\mathrm{TN}}},$$

such that the total cost $c_{\mathrm{QC}}$ of the study with RTQC is

(17) $$c_{\mathrm{QC}}=N_{\mathrm{recr}}c_{\mathrm{recr}}+N_{\mathrm{scan}}c_{\mathrm{scan}}=N_{\mathrm{target}}\frac{{c_{\mathrm{recr}}(p}_{\mathrm{TN}}+p_{\mathrm{FN}})+c_{\mathrm{scan}}}{p_{\mathrm{TN}}}=N_{\mathrm{target}}\frac{c_{\mathrm{recr}}((1-p_{N})LR^{-}+p_{N})+\frac{c_{\mathrm{scan}}}{\mathrm{spec}}}{p_{N}}.$$

The relative cost of applying RTQC over not applying RTQC is given by

(18) $$c_{\mathrm{rel}}=\frac{c_{\mathrm{QC}}}{c_{\mathrm{base}}},$$

where values below one is favorable. We can express $c_{\mathrm{rel}}$ as a function with respect to sensitivity, specificity, negative rate $p_{N}$ and relative recruitment cost α, namely

(19) $$c_{\mathrm{rel}}\left(\mathrm{spec},\mathrm{sens};\alpha ,p_{N}\right)=\alpha \left(\left(1-p_{N}\right)LR^{-}+p_{N}\right)+\left(1-\alpha \right)\frac{1}{\mathrm{spec}}.$$

The relative recruitment cost is defined by $\alpha ≔\frac{c_{\mathrm{recr}}}{{c_{\mathrm{recr}}+c}_{\mathrm{scan}}}\in [0,1]$. The savings due to applying RTQC in a clinical study can be quantified as

(20) $$\;\mathrm{savings}=1-c_{\mathrm{rel}}.$$

#### 4.4.3 Adjusting sensitivity and specificity of the image quality assessment

From the expressions above, the negative rate $p_{N}$ is given by the underlying data quality and depends on the operator, the imaging system, the image formation, and other environmental influences, but is unaffected by our RTQC. However, we can influence $E\left[\mathrm{usability}\right]$, $E\left[\mathrm{\#scans}\right]$ and ultimately $c_{\mathrm{rel}}$ by modifying sensitivity (sens) and specificity (spec) of the IQA. As explained in Section C.4., the relationship between sens and spec defined by the ROC curve can be seen as a function; sens=ROC(1−spec) and $\mathrm{spec}=1-\mathrm{RO}C^{-1}(\mathrm{sens})$. Therefore, adjusting the CBD of the SVM can modify the computations of our quality control. Therefore, adjusting the CBD of the SVM can modify the computation of the RTQC.

#### 4.4.4 Optimal quality control

To establish an effective and efficient RTQC tool, our goal is to maximize the usability of the data, E[usability], for each recruited patient, while minimizing extra costs and burden (which can be included in the scanning cost) on each patient caused by measurements (E[#scans]). Achieving this balance is done by minimizing the relative cost $c_{\mathrm{rel}}$ (Eq.(19)), with

(21) $$c_{\mathrm{rel}}\left(\mathrm{spec},\mathrm{sens};\alpha ,p_{N}\right)=\alpha \left(\left(1-p_{N}\right)LR^{-}+p_{N}\right)+\left(1-\alpha \right)\frac{1}{\mathrm{spec}}.$$

For a given cost ratio α, negative ratio $p_{N}$ and ROC curve, the cost-optimal sensitivity is determined by solving the one-dimensional optimization problem

(22) $$\mathrm{sen}s^{*}=\underset{\mathrm{sens}\in [0,1]}{\arg\min}\;\alpha \left(\frac{\left(1-p_{N}\right)(1-\mathrm{sens})}{1-\mathrm{RO}C^{-1}(\mathrm{sens})}+p_{N}\right)+\frac{\left(1-\alpha \right)}{1-\mathrm{RO}C^{-1}(\mathrm{sens})}.$$

Then, the CDB of the IQA (SVM) for the optimal quality control can be derived to match the resulting optimal sensitivity $\mathrm{sen}s^{*}$.

### CRediT authorship contribution statement

**Hubert Preißl:** Resources, Project administration, Methodology, Investigation, Data curation. **Andreas L. Birkenfeld:** Resources, Project administration, Methodology, Investigation, Funding acquisition. **Nikoletta Katsouli:** Investigation, Data curation. **Nikolina-Alexia Fasoula:** Writing – review & editing, Project administration, Methodology, Investigation, Data curation. **Angelos Karlas:** Writing – review & editing, Supervision, Resources, Project administration, Methodology, Investigation, Data curation. **Michael Kallmayer:** Supervision, Resources, Project administration, Methodology, Investigation. **Daniela Branzan:** Writing – review & editing, Resources, Project administration. **María Begoña Rojas López:** Writing – review & editing, Writing – original draft, Visualization, Validation, Software, Methodology, Investigation, Formal analysis, Data curation, Conceptualization. **Anette-Gabriele Ziegler:** Writing – review & editing, Resources, Project administration. **Manuel Gehmeyr:** Writing – review & editing, Writing – original draft, Visualization, Validation, Software, Methodology, Investigation, Formal analysis, Data curation, Conceptualization. **Dominik Jüstel:** Writing – review & editing, Validation, Supervision, Project administration, Methodology, Investigation, Formal analysis, Conceptualization. **Suhanyaa Nitkunanantharajah:** Writing – review & editing, Validation, Supervision, Methodology, Conceptualization. **Vasilis Ntziachristos:** Writing – review & editing, Writing – original draft, Supervision, Resources, Project administration, Methodology, Funding acquisition, Conceptualization. **Andreas Vosseler:** Resources, Project administration, Methodology, Investigation, Data curation. **Reiner Jumpertz von Schwartzenberg:** Resources, Project administration, Methodology, Investigation, Funding acquisition.

## Ethics declaration

Written informed consent to take part in the study and to publish the article has been obtained from all participants or their legal representatives. The privacy rights of participants have been observed.

This study was performed in compliance with relevant laws, regulatory frameworks and guidelines where the research took place. This study was approved by the 1–2) Ethics Committee of the Technical University of Munich; 3) Ethics Committee of the University Hospital of Tübingen. (Approval No. 1) 109/17 S; 2) 326/19 S; 3) NCT01947595).

## Inclusion & ethics statement

All authors included in this work have fulfilled the authorship criteria required by Nature Portfolio journals. Their contributions were essential to the conceptualization and development of the RTQC framework, algorithm implementation, data acquisition, development of the clinical cost-saving estimation framework, and the analysis and interpretation of the results.

The data used for training, validation, and analysis of the algorithms were acquired as part of three clinical studies conducted at two institutions in Germany: the TUM University Hospital (Hospital Rechts der Isar), Technical University of Munich (TUM), and the University Hospital Tübingen.

All studies were conducted in accordance with the ethical principles of the Declaration of Helsinki for medical research involving human participants. All participants freely provided written informed consent.

Ethical approval for the two clinical studies in the TUM University Hospital (Hospital Rechts der Isar) from Technical University of Munich were obtained from Ethics Committees in the Technical University Munich with the following protocol IDs: Diabetes study: 109/17 S and CVD study: 326/19 S.

The clinical study conducted by Klinikum University Tübingen was the Prediabetes Lifestyle Interventional Study [57] (PLIS), registered under ClinicalTrials.gov with ID NCT01947595, and the Ethics Committee at the University Hospital of Tübingen approved the study protocol.

## Declaration of Generative AI and AI-assisted technologies in the writing process

During the preparation of this work, the authors used ChatGPT (OpenAI) to assist with language editing, including improving clarity, sentence structure, and organization of the manuscript. The authors reviewed and edited all AI-assisted content and take full responsibility for the content of the published article.

## Declaration of Competing Interest

The authors declare the following financial interests/personal relationships which may be considered as potential competing interests: V.N. is a founder and equity owner of iTheraNEXT Imaging GmbH, Maurus OY, sThesis GmbH, Spear UG, Biosense Innovations P.C. and I3 Inc.

## Data Availability

Data will be made available on request.
