# Supplementary material for: Real-time quality control in optoacoustic mesoscopy for enhancing data quality and standardization in clinical studies
Source: Photoacoustics. 2026 Aug 27;51:100878. doi: 10.1016/j.pacs.2026.100878 (PMC13563549; doi:10.1016/j.pacs.2026.100878)
Supplement: Supplementary file 1 [file mmc1.docx]

# Supplementary Information

### 1.1 Supplementary Materials

#### 1.1.1 Selection of best classifier

We evaluated multiple classifiers to determine the most appropriate model for our binary classification task. To identify the best classifier, we employed a patient-wise leave-one-out cross validation on the training data set. The classifiers trained and evaluated included linear, polynomial, and Gaussian SVM, Naïve Bayes model, k-nearest neighbor, and a fully connected neural network (FCNN). All the models were implemented in MATLAB and their performances are shown in Supplementary Figure 1 and Supplementary Table 1. For each model, we used normalized data and applied an asymmetric cost function as described in Methods 4.3.3 for the best performing classifier.

The models’ performances were evaluated according to the maximum g-mean of sensitivity and specificity along the ROC curve of the classifiers (Methods 4.3.3), as indicated by a yellow dot on the ROCs in Supplementary Figure 1. The SVM with a third-degree polynomial kernel was identified as the best performing model, as it achieved the highest maximum g-mean among all considered models and was therefore chosen for the development of the IQA tool.

#### 1.1.2 Analysis of the data RTQC tool

If all discarded scans can be revisited and checked by experts to determine their “true quality”, false positives can be reintegrated. Consequently, the usability, defined as the expected value of obtaining at least one HQ scan, increases to

|  | $E\left[ usability^{*} \right]=1-p_{FN}\sum_{j=0}^{\infty} {(p}_{TP})^{j}=\frac{p_{N}}{1-p_{TP}}=\frac{p_{N}}{1-\left( 1-p_{N} \right)sens}.$ | $(23)$ |
| --- | --- | --- |

However, this process is time consuming and expensive as experts are needed for IQA. Hence, the adapted usability score will not be considered in the main manuscript.

**Supplementary Table 1. Prediction performance of the classification models evaluated using patient-wise leave-one-out cross validation**. The SVM with third-degree polynomial kernel achieved the highest maximum geometric mean (g-mean). AUC: area under the receiver operating characteristic (ROC) curve, SVM: support vector machine, FCNN: Fully Connected Neural Network

| Model | AUC | Max g-mean | Specificity max gmeans | Sensitivity max gmeans |
| --- | --- | --- | --- | --- |
| SVM linear | 0.759 | 0.688 | 0.728 | 0.651 |
| SVM polynomial order 2 | 0.779 | 0.720 | 0.766 | 0.676 |
| SVM polynomial order 3 | 0.783 | 0.726 | 0.830 | 0.636 |
| SVM polynomial order 4 | 0.773 | 0.723 | 0.796 | 0.657 |
| SVM Gaussian | 0.766 | 0.720 | 0.770 | 0.674 |
| SVM perceptron | 0.621 | 0.613 | 0.682 | 0.551 |
| Naïve Bayes | 0.757 | 0.692 | 0.759 | 0.658 |
| k-nearest neighbor | 0.771 | 0.700 | 0.705 | 0.695 |
| FCNN | 0.770 | 0.701 | 0.759 | 0.658 |

**Supplementary Figure 1. Accuracy of our image quality assessment (IQA) classifier across different image qualities.** Bins represent the classification accuracy of the IQA for all test images with a mean opinion score (MOS) falling within each bin’s boundaries. The decision boundary separating suboptimal quality (SQ) from high quality (HQ) is marked at an MOS of 2.5.

**Supplementary Figure 2.** Example RSOM images together with their corresponding expert-assigned mean opinion score (MOS), predicted quality labels, and quality metrics. **a.** High quality (HQ) scan. **b.** HQ scan with moderate noise. **c.** Scan close to the binarization threshold (BT); true negative (TN) example labelled and classified as HQ. **d.** Scan close to the binarization threshold (BT); true positive (TP) example labelled and classified as suboptimal quality (SQ). **e.** Scan with reduced vascular visibility. **f.** Scan with excessive noise. **g.** Scan with poor acoustic coupling, likely caused by air bubbles in the coupling medium. **h.** Scan with severe motion distortion.
